# Dimethylarginine Dimethylaminohydrolase 2 (*DDAH 2*) Gene Polymorphism, Asymmetric Dimethylarginine (ADMA) Concentrations, and Risk of Coronary Artery Disease: A Case-Control Study

**DOI:** 10.1038/srep33934

**Published:** 2016-09-28

**Authors:** Chao Xuan, Long-Qiang Xu, Qing-Wu Tian, Hui Li, Qing Wang, Guo-Wei He, Li-Min Lun

**Affiliations:** 1 Department of Clinical Laboratory, The Affiliated Hospital of Qingdao University, Qingdao, China; 2 Department of Surgery, TEDA International Cardiovascular Hospital, Tianjin & The Affiliated Hospital of Hangzhou Normal University, Hangzhou, China; 3 Department of Surgery, Oregon Health and Science University, Portland, Oregon

## Abstract

Asymmetric dimethylarginine (ADMA) has been shown to be an independent predictor of cardiovascular diseases. Dimethylarginine dimethylaminohydrolase 2 (DDAH 2) promotes the metabolism of ADMA and plays a key role in the regulation of acute inflammatory response. With the present study, we investigated the relationship between *DDAH 2* polymorphisms and risk of coronary artery disease (CAD) and its association to plasma ADMA concentrations. We used the haplotype-tagging SNP approach to identify tag SNPs in *DDAH 2*. The SNPs were genotyped by PCR and sequenced in 385 CAD patients and 353 healthy controls. Plasma concentrations of ADMA were determined using enzyme-linked immunosorbent assay (ELISA). A promoter polymorphism −449C/G (rs805305) in *DDAH 2* was identified. Compared with the ADMA concentrations in CC genotype (0.328 ± 0.077 μmol/l), ADMA concentrations in CG + GG genotype were significantly increased (0.517 ± 0.090 μmol/l, *P* < 0.001). No significant associations between the −449C/G and risk of CAD were detected in the genetic models. The results of this study suggest that Genetic −499C/G polymorphism in *DDAH 2* gene may affect the plasma ADMA concentrations in patients with CAD. However, it does not indicate a novel genetic risk marker for CAD.

A major cause of the endothelial dysfunction is decreased bioavailability of nitric oxide (NO), a potent biological vasodilator synthesized in vascular endothelium from L-arginine by the action of endothelial NO synthase (eNOS)^1^,^2^. Asymmetric dimethylarginine (ADMA) is produced in human cells during proteolysis of methylated nuclear proteins. It acts as an endogenous inhibitor of eNOS by competing with L-arginine, and this in turn causes endothelial dysfunction and vascular disease^3^,^4^.

Although a proportion of ADMA is excreted in the urine, it has been estimated that more than 70% of ADMA is metabolized by the enzyme dimethylarginine dimethylaminohydrolase (DDAH) *in vivo*^5^. Two isoforms of DDAH have been identified and they are each encoded by genes positioned on chromosomes 1p22 and 6p21.3^6^. DDAH 1 is typically found in tissues expressing neuronal NOS, whereas DDAH 2 predominates in tissues containing the endothelial isoform of NOS^7^. Although the apparent rate of ADMA metabolism for DDAH 2 is almost 70 times less than that of DDAH-1, *DDAH-2* gene silencing also reduced endothelial dependent relaxation by 40% *in vivo*^8^. Recent studies established a marked cellular disparity in the expression of the two isoforms, with DDAH 2 making a predominant contribution in endothelial cells where it determines NO bioactivity^9^.

The aim of our study was to investigate the relationship between the *DDAH 2* polymorphisms and risk of coronary artery disease (CAD) and its association to plasma ADMA concentrations in a Chinese population.

## Materials and Methods

### Subjects

This research protocol was approved by the Institutional Review Board of The Affiliated Hospital of Qingdao University, and the experiments on human subjects were performed in accordance with relevant guidelines and regulations. 385 CAD patients (255 males and 130 females, aged 32–78 years) and 353 control subjects (232 males and 121 females, aged 30–76 years) were recruited from the Affiliated Hospital of Qingdao University in September, 2013–March, 2016. Participants were included if they met at least one of the following three inclusion criteria: (1) Chest pain with electrocardiogram (ECG) changes and/or elevated CK-MB or cardiac troponin T/I (cTn T/I); (2) Angiographically verified CAD; (3) Stable or unstable angina along with positive Treadmill Test (TMT) or ECG ST-T changes with elevated CK-MB or cTn T/I. Patients with other severe medical conditions, pregnant women, and lactating mothers were excluded from the study. Controls for the study were sampled during the same time period. Controls were healthy volunteers, free of any signs or symptoms of cardiovascular disease. They were recruited from a geographic background similar to that of the patients and came from community samples or hospital staff. All subjects provided written informed consent prior to participation and also consented to having blood drawn at the time of angiography or time of screening for DNA extraction.

### Anthropometric and Clinical Parameters

A full physical examination of all the subjects was carried out. Height and weight were recorded to the nearest 0.5 cm and 0.1 kg, respectively. The body mass index (BMI) was calculated using the formula kg/m^2^. Blood pressure was measured twice by an examining physician at an interval of 30 min using automated oscillometric device. An average value of the two readings served as the final measure of blood pressure. A diagnosis of hypertension was based on the presence of elevated systolic (≥140 mmHg) and/or diastolic (≥90 mmHg) blood pressure, or current use of antihypertensive medications. Diabetes was diagnosed when the subject met at least one of the following three criteria: 1) a random venous plasma glucose concentration ≥11.1 mmol/l; 2) a fasting plasma glucose concentration ≥7.0 mmol/l; 3) two hour plasma glucose concentration ≥11.1 mmol/l (two hours after 75 g anhydrous glucose in an oral glucose tolerance test).

### Biochemical Measurements

Blood samples were drawn from all participants after overnight fasting of at least 8 hours. Serum levels of fasting blood glucose (FBG), triglyceride (TG), total cholesterol (TCHO), low-density lipoprotein cholesterol (LDL-C), and high-density lipoprotein cholesterol (HDL-C) were determined using an automatic biochemistry analyzer (Hitachi HCP-7600, Japan).

### Plasma ADMA Measurement

2 ml of venous blood from patients or healthy participants was obtained after the overnight fast before the angiography. The blood samples were centrifuged at 1,550 g for 20 min via the Centrifuge 5810R (Eppendorf, Germany). The serum was stored at −80 °C for enzyme-linked immunoassay (ELISA). Serum concentrations of ADMA were determined by ELISA (Cloud-Clone Corp, Houston, TX, USA). This assay employs the competitive inhibition enzyme immunoassay technique. A monoclonal antibody specific to ADMA has been pre-coated onto a microplate. A competitive inhibition reaction was launched between biotin labeled ADMA and unlabeled ADMA (standards or samples) with the pre-coated antibody specific to ADMA. After incubation, the unbound conjugate was washed off. Avidin conjugated to horseradish peroxidase (HRP) was then carefully added to each microplate and incubated. The amount of bound HRP conjugate was inversely proportional to the concentration of ADMA in the sample. After addition of the substrate solution, the intensity of color developed was inversely proportional to the concentration of ADMA in the sample. The inter- and intra-assay variations were 5.4% and 3.8%, respectively.

### DNA Isolation, Genetic Variant Selection and Genotyping

Genomic DNA was isolated from whole blood samples using the QIAamp DNA Blood Mini Kit (QIAGEN, Valencia, CA, USA) as per the instructions given by the manufacturer. DNA was extracted from 200 μl of whole blood using the spin columns provided. The isolated DNA was stored at −20 °C.

**Figure 1:**
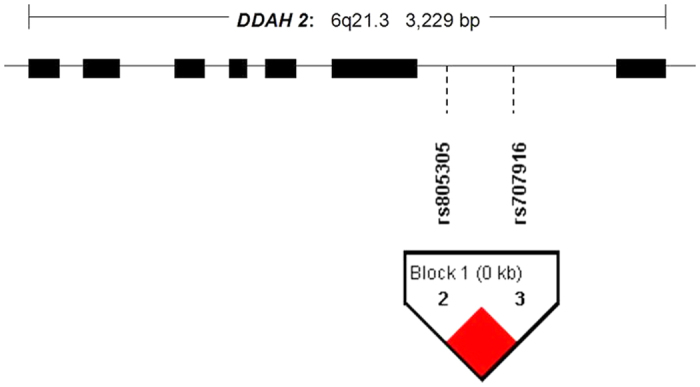
LD of the SNP markers in *DDAH 2* in the HapMap CHB (Chinese Han in Beijing) population. A standard scheme is used to display LD with a solid black diamond for absolute LD (r^2^ = 1).

To better cover the common variations across the *DDAH 2*, the selection of genetic variants was based on the HapMap CHB (Chinese Han in Beijing) sample using the pairwise option of the Haploview version of the Tagger program. A minimum r^2^ = 0.8 was chosen as a threshold for all analyses. Only one tag SNP (rs707916) with a minor allele frequency >5% was genotyped within a 3.5-kb region spanning *DDAH 2* on chromosome 6p21.3. A promoter variant −449C/G (rs805305) in *DDAH 2* was in complete linkage disequilibrium with rs707916 (Fig. 1) and was selected for genotyping in all of the subjects studied since it has been reported to be associated with changes in plasma ADMA concentrations^10^,^11^.

The primers for *DDAH 2* −449C/G (rs805305) polymorphism was: forward: 5′-GCGGAGAGAGGATGCTTAAC-3′ and reverse: 5′-ACACCTGTTGCCCCTGCT-3′. PCR conditions consisted of one cycle of 10 min at 95 °C, 36 cycles of 30 sec at 94 °C and 1 min at 65 °C, followed by 30 min at 72 °C in GeneAmp PCR 2720 (Applied Biosystems, Foster City, CA). Direct sequencing of the PCR product was performed by a genomic company (Genewiz Biotechnology Co., Ltd., Suzhou, China), GeneTools software (Gene Tools, LLC, Philomath, OR, USA) was used to identify the SNPs. The position of the nucleotide sequence was based on the reference sequence obtained from the National Center for Biotechnology Information (NCBI) nucleotide database. The SNP database hosted by NCBI database was used (http://www.ncbi.nlm.nih.gov/SNP/).

### Statistical Analysis

Values are means ± standard deviation (SD) unless otherwise specified. The distributions of the categorical variables were expressed as frequencies and percentages, and the comparisons calculated by using chi-square test or Fisher exact test, as appropriate. Comparisons between groups for study variables were done using the unpaired student’s t test for normally distributed parameters. *HWE* (Hardy–Weinberg equilibrium) was calculated using a *Q* test with one degree of freedom^12^. We examined the contrast of the GG vs. CC, GG vs. CG and also examined the dominant genetic model (CC + CG vs. GG) and the recessive genetic model (CC vs. CG + GG)^13^,^14^. The association between *DDAH 2 −*499C/G polymorphism and risk of CAD was estimated by calculating odds ratio (OR) and its 95% confidence interval (CI). Multivariate unconditional logistic regression was used to estimate ORs and 95% CIs after adjustment for age, gender, BMI, HDL-C, LDL-C, TCHO, TG, FBG, hypertension, diabetes, and smoking.

All reported *P* values are two-sided, and *P* < 0.05 was considered statistically significant. Analyses were performed using SPSS software version 11.0 (SPSS, Chicago, USA) and Stata software version 10.0 (StataCorp, Texas, USA).

## Results

### Clinical Characteristics of Participants

A total of 385 CAD patients (mean age 64.21 ± 8.69; 77.1% men) and 353 controls (mean age 64.16 ± 8.58; 74.8% men) took part in the study. Clinical characteristics of all participants at baseline are summarized in Table 1. Overall, CAD patients had higher BMI, FBG, LDL-C, TG, ADMA, and higher prevalence of hypertension compared with controls (all *P* < 0.05). There were no differences between the groups with respect to age, gender, TCHO, HDL-C, diabetes, and smoking.

### Polymorphism of *DDAH 2* gene

The SNP of *DDAH 2* [rs805305 (−449C/G)] was genotyped in 385 CAD patients and 353 controls. The genotypes of the SNP examined in CAD patients and controls are summarized in Table 2. The distribution of the *DDAH 2* genotype in the controls was compatible with *HWE* (*P* > 0.05), with allele frequencies of 39.24% and 60.76% for the C and G alleles, respectively.

### Association Between *DDAH 2* Gene Polymorphism and Risk and CAD

The distribution of allele and genotype frequencies is shown by group in Table 2. We did not detect significant associations between *DDAH 2* −449C/G polymorphism and risk of CAD in genetic models for GG vs. CC (OR = 0.796, 95% CI: 0.522–1.215), GG vs. GC (OR = 0.979, 95% CI: 0.711–1.348), dominant genetic model (OR = 0.925, 95% CI: 0.68–1.248), and recessive genetic model (OR = 0.806, 95% CI: 0.550–1.182).

Similar results were obtained after adjusting for confounding factors such as age, gender, BMI, HDL-C, LDL-C, TCHO, TG, FBG, hypertension, diabetes, and smoking. The associations were not significant for GG vs. CC (OR = 0.697, 95% CI: 0.406–1.195; adjusted *P* = 0.189), GG vs. GC (OR = 1.141, 95% CI: 0.808–1.612; adjusted *P* = 0.454), dominant genetic model (OR = 0.839, 95% CI: 0.572–1.232; adjusted *P* = 0.371), and recessive genetic model (OR = 0.846, 95% CI: 0.576–1.243; adjusted *P* = 0.394). See Table 3 for a list of the main results.

### *DDAH 2* Gene Polymorphism and ADMA Concentrations

**Figure 2:**
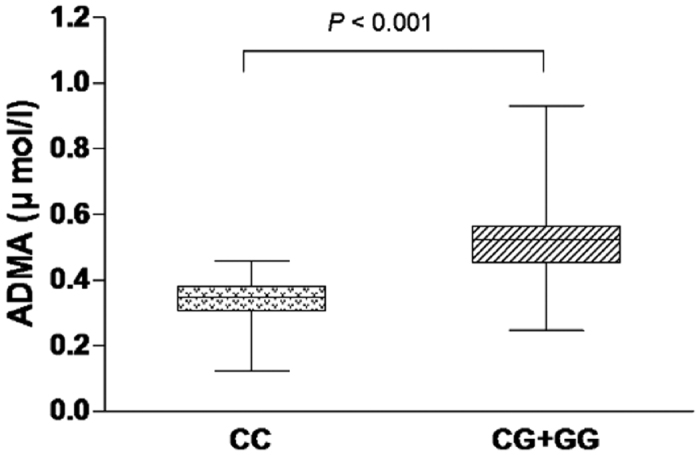
Influence of the *DDAH 2* gene polymorphism on ADMA concentrations in CAD patients. The ADMA concentrations in CG + GG genotypes of *DDAH 2 gene* −499C/G polymorphism (0.328 ± 0.077 μmol/l) shows significantly increase to compare with the concentrations in CC genotype (0.517 ± 0.090 μmol/l, *P* < 0.001, unpaired *t* test).

There was a trend towards increasing ADMA between different *DDAH 2* genotypes. ADMA was most abundant in the GG homozygotes, least abundant in the CC homozygotes and detectable at intermediate levels in the heterozygotes. When compared the ADMA concentrations in CC genotype (0.328 ± 0.077 μmol/l), the ADMA concentrations in CG + GG genotype were significantly increased (0.517 ± 0.090 μmol/l, *P* < 0.001, unpaired *t* test; Fig. 2).

## Discussion

With the present study, we demonstrated that (1) the genetic polymorphism (−499 C/G rs 805305) in the *DDAH 2* genes was significantly associated with plasma ADMA concentrations in participants with CAD, but (2) the polymorphism may not be related to the risk of CAD in this Chinese population.

ADMA as an endogenous inhibitor of nitric oxide synthase (NOS) was characterized in the 1992 study by Vallance and coworkers^15^. ADMA is synthesized when arginine residues in proteins are methylated by the action of protein arginine methyltrans ferases. Humans generate approximately 300 μmol of ADMA per day^16^. The clearance of ADMA from the body is either by excretion in the urine or by metabolism. A number of cells that generate ADMA can also inactivate it by metabolizing it to citrulline, in a reaction catalyzed by the enzyme DDAH^17^. ADMA inhibits the three isoforms of NOS and is equipotent with L-NMMA. High levels of circulating ADMA have been shown to impair NO dependent functions in the vascular wall. It can also uncouple the enzyme, generate super-oxides, and it has been linked to endothelial dysfunction^18^. In previous studies, we demonstrated that the increased ADMA levels directly cause endothelial dysfunction by down-regulating mRNA and protein expression of eNOS, decreasing the generation of NO, increasing superoxide production, and inducing endothelial apoptosis in human internal mammary arteries as well as in porcine coronary arteries^19^,^20^.

Numerous studies have demonstrated a relationship between high ADMA concentrations and cardiovascular diseases^21^. Elevated ADMA has a high prevalence in hypercholesterolemia, hyperhomocysteinemia, diabetes mellitus, peripheral arterial occlusive disease, hypertension, chronic heart failure, and other diseases^22^,^23^,^24^,^25^,^26^,^27^. In our recent meta-analysis involving 2,939 CAD patients and 1,774 controls, we found that high ADMA levels are a risk factor in patients with CAD (*P* = 1.16 e–7). The subgroup analysis also indicated that increased ADMA levels were detected in different clinical types of CAD, including myocardial infarction (*P* = 0.006), stable angina pectoris (*P* = 0.020), and unstable angina pectoris (*P* = 0.003)^28^. With the present results, we also demonstrate significantly higher plasma ADMA concentrations in CAD patients (0.481 ± 0.115 μmol/l) compared to controls (0.459 ± 0.090 μmol/l, *P* = 0.003).

ADMA is actively metabolized by DDAH, which is expressed as two isoforms. The major difference between the two isoforms is tissue expression pattern. DDAH 1 is widely expressed, especially in liver and kidney at sites of NOS expression. DDAH 2 is expressed at relatively high levels in all fetal tissues, while in adults, concentrations fall and sites of expression become more selective. DDAH 2 predominates in the vascular endothelium, which is the site of eNOS expression^9^. *DDAH 2* localizes to 6p21.3, a region which contains many genes involved in the immune and inflammatory responses, and has been linked with susceptibility to several autoimmune diseases. This localization and its wide expression in immune cells suggest that *DDAH 2* has the potential to be a disease-susceptibility gene^6^. However, the role of DDAH 2 in ADMA metabolism and cardiovascular disease remains controversial. Wang and co-workers demonstrated that *DDAH 2* gene silencing had no effect on plasma ADMA in a rat sample^29^. In contrast, results of other studies indicated that *DDAH 2* genetic variations were significantly associated with serum ADMA concentrations, especially in human samples^10^,^11^. We present the novel finding that the CAD patients carrying the G alleles of *DDAH 2* gene −499 C/G have higher plasma ADMA concentrations.

The *DDAH 2* gene −499 C/G polymorphism has been linked with the risk of a number of cardiovascular diseases, including type 2 diabetes, hypertension, intracerebral hemorrhage, and hemodynamic shock among others^30^,^31^,^32^. However, there are comparatively few studies, most with small sample sizes, examining the relationship between the polymorphism and risk of CAD. GAD and colleagues demonstrated that the G allele of *DDAH 2* gene −499 C/G polymorphism is an important risk factor in male 35–50 year-old Egyptian CAD patients (100 CAD patients vs. 100 healthy controls)^33^. However, Xu and colleagues indicated that no association was observed between the *DDAH 2* polymorphisms and risk of CAD (180 Chinese CAD patients vs. 180 healthy controls)^34^. The present study evaluated the association between the polymorphism and risk of CAD in a large sample of 738 participants (385 cases and 353 controls). We failed to find a significant association in this Chinese population. Similar results were obtained after adjusting for confounding factors such as age, gender, BMI, HDL-C, LDL-C, TCHO, TG, FBG, hypertension, diabetes, and smoking. To our knowledge, *DDAH 2* not only directly affects plasma ADMA concentrations, but also has an important role in cellular differentiation or cell cycle control processes in which NO is involved^35^,^36^. Future studies should address other mechanisms of *DDAH 2* gene with regard to the risk of CAD, including methylation.

In conclusion, genetic polymorphism (−499C/G, rs805305) in *DDAH 2* gene was found to be significantly associated with plasma ADMA concentrations in patients with CAD. However, our findings suggest that the *DDAH 2* variant is not a novel genetic risk marker for CAD.

## Additional Information

**How to cite this article**: Xuan, C. *et al*. Dimethylarginine Dimethylaminohydrolase 2 (*DDAH 2*) Gene Polymorphism, Asymmetric Dimethylarginine (ADMA) Concentrations, and Risk of Coronary Artery Disease: A Case-Control Study. *Sci. Rep.* **6**, 33934; doi: 10.1038/srep33934 (2016).

## Figures and Tables

**Table 1:** General Characteristics of Study Population Including Controls and CAD Patients.

	Control (n = 353)	CAD (n = 385)	*P-Value*
Age (years) *	64.16 ± 8.58	64.21 ± 8.69	0.932
Sex, n (male/female) #	264/89	297/88	0.454
BMI (kg/m^2^) *	22.55 ± 2.88	24.22 ± 3.50	0.000
FBG (mmol/l) *	5.27 ± 1.28	5.48 ± 1.44	0.038
TCHO (mmol/l) *	4.88 ± 0.64	4.92 ± 0.84	0.499
HDL-C (mmol/l) *	1.38 ± 0.31	1.36 ± 0.37	0.235
LDL-C (mmol/l) *	2.90 ± 0.49	3.04 ± 0.65	0.001
TG (mmol/l) *	1.67 ± 0.49	1.76 ± 0.58	0.029
Hypertension, n (%) #	125 (35.41)	168 (43.63)	0.023
Diabetes, n (%) #	46 (13.03)	70 (18.18)	0.055
Smoking, n (%) #	131 (37.11)	120 (31.17)	0.089
ADMA (μmol/l) *	0.459 ± 0.090	0.481 ± 0.115	0.003

BMI: body mass index; SBP: systolic blood pressure; DBP: diastolic blood pressure; FBG: fasting blood glucose; TCHO: total cholesterol; HDL: high-density lipoprotein cholesterol; LDL: low-density lipoprotein cholesterol; TG: triglyceride.

^*^Continuous variables are expressed as mean ± SD. The *P*-value of the continuous variables was calculated by the unpaired *t* test.

^#^Categorical variables are expressed as percentages. The *P*-value of the categorical variables was calculated by χ^2^ test.

**Table 2:** Genotype Frequency rs805305 of *DDAH 2* gene in CAD and Control Groups.

Groups	Genotype (%)			C allele (%)	G allel (%)	*HWE*
	CC	CG	GG			
Control (n = 353)	56 (15.86)	165 (46.74)	132 (37.39)	39.24	60.76	0.738
CAD (n = 385)	73 (18.96)	175 (45.45)	137 (35.78)	41.69	58.31	—

CAD, Coronary Artery Disease; *HWE*: Hardy–Weinberg Equilibrium.

**Table 3:** Association Between the −449C/G Variant in *DDAH 2* Gene and Risk of CAD.

Genetic Models	Crude OR (95% CI) *	Adjusted OR (95% CI)**	Adjusted *P*-value
GG vs. CC	0.796 (0.522–1.215)	0.697 (0.406–1.195)	0.189
GG vs. CG	0.979 (0.711–1.348)	1.141 (0.808–1.612)	0.454
Dominant genetic model #	0.925 (0.685–1.248)	0.839 (0.572–1.232)	0.371
Recessive genetic model †	0.806 (0.550–1.182)	0.846 (0.576–1.243)	0.394

^*^Crude ORs were calculated with 2 × 2 cross-tabulation.

^**^Adjusted ORs were obtained from multivariate logistic regression after controlling for age, gender, BMI, HDL-C, lDL-C, TCHO, TG, FBG, hypertension, diabetes, and smoking.

^#^Dominant genetic model (CC + CG vs. GG).

^†^Recessive genetic model (CC vs. CG + GG).
